# Removal of 8-oxo-GTP by MutT hydrolase is not a major contributor to transcriptional fidelity

**DOI:** 10.1093/nar/gku912

**Published:** 2014-10-07

**Authors:** Alasdair J.E. Gordon, Dominik Satory, Mengyu Wang, Jennifer A. Halliday, Ido Golding, Christophe Herman

**Affiliations:** 1 Department of Molecular and Human Genetics, Baylor College of Medicine, Houston, TX 77030, USA; 2 Verna and Marrs McLean Department of Biochemistry and Molecular Biology, Baylor College of Medicine, Houston, TX 77030, USA; 3 Graduate Program in Structural and Computational Biology and Molecular Biophysics, Baylor College of Medicine, Houston, Texas, 77030, USA; 4 Dan L. Duncan Cancer Center, Baylor College of Medicine, Houston, Texas 77030, USA; 5 Department of Molecular Virology and Microbiology, Baylor College of Medicine, Houston, Texas 77030, USA

## Abstract

Living in an oxygen-rich environment is dangerous for a cell. Reactive oxygen species can damage DNA, RNA, protein and lipids. The MutT protein in *Escherichia coli* removes 8-oxo-deoxyguanosine triphosphate (8-oxo-dGTP) and 8-oxo-guanosine triphosphate (8-oxo-GTP) from the nucleotide pools precluding incorporation into DNA and RNA. While 8-oxo-dGTP incorporation into DNA is mutagenic, it is not clear if 8-oxo-GTP incorporation into RNA can have phenotypic consequences for the cell. We use a bistable epigenetic switch sensitive to transcription errors in the *Escherichia coli lacI* transcript to monitor transient RNA errors. We do not observe any increase in epigenetic switching in *mutT* cells. We revisit the original observation of partial phenotypic suppression of a *lacZ*_amber_ allele in a *mutT* background that was attributed to RNA errors. We find that Lac^+^ revertants can completely account for the increase in β-galactosidase levels in *mutT lacZ*_amber_ cultures, without invoking participation of transient transcription errors. Moreover, we observe a fluctuation type of distribution of β-galactosidase appearance in a growing culture, consistent with Lac^+^ DNA revertant events. We conclude that the absence of MutT produces a DNA mutator but does not equally create an RNA mutator.

## INTRODUCTION

Errors in information transfer from DNA to RNA to protein are inevitable. Transcription errors occur at a rate of ∼10^−5^ per residue in *Escherichia coli* (1,2), over 10 000× higher than errors in DNA synthesis. Errors in DNA synthesis can produce heritable change in phenotype due to alteration of protein function. Studies that have focused on the mechanisms of DNA replication and repair in *E. coli* have provided a major framework for understanding the fidelity of genetic transmission from cell to cell and have revealed a series of fidelity mechanisms responsible for maintaining DNA integrity (3). Transcription errors, although transient in nature, can also have phenotypic consequences for the cell, including transient (4) and heritable phenotypic change (5,6). Unlike DNA fidelity, the mechanisms ensuring transcription fidelity *in vivo* are not well characterized due to the difficulty of isolating such transient errors in mRNA (7). We have harnessed the classical bistable switch in the *lac* operon, a memory-module, to capture and monitor the consequences of transient transcription errors in living *E. coli* cells, providing an appropriate tool to study proteins involved in modulating RNA fidelity (5,6).

The *lac* operon comprises an autocatalytic positive feedback loop allowing a heritable all-or-none epigenetic switch at a maintenance concentration of inducer [that concentration of inducer which does not activate transcription of the operon but allows an already induced cell to remain induced (5,8–9)]. The *lac* repressor is rare (∼5 tetramers per cell) (10). A transient depletion of repressor within a cell will lead to a transient derepression of the operon, producing a burst of *lacY* permease gene expression (11). At the maintenance concentration of the nonmetabolizable inducer thio-methylgalactoside (TMG), this burst of permease will trigger an autocatalytic positive feedback response, so that the new induced state will be heritably maintained through cell division in a clonal cell population (5,6).

Using single-cell analysis, we have previously shown that the frequency of epigenetic switching from the OFF expression state to the ON expression state of the *lac* operon is increased when the fidelity of RNA transcription is decreased due to error-prone RNA polymerases, error-prone transcription sequences or to the absence of auxiliary RNA fidelity factors GreA and GreB (functional analogues of eukaryotic TFIIS) (5,6). In addition, the 1000-fold difference between the genetic mutation frequency (*lacI*^+^ to *lacI*^−^) and the epigenetic switch frequency observed in the wild-type strain (5) demonstrates that this epigenetic switch system is not affected by mutation, even the increased mutagenesis seen in mutator strains (5).

Treffers was the first to discover a mutator strain of bacteria (12). His *mutT* allele facilitated a unique unidirectional mutational signature, A:T → C:G transversions (13). It was later shown that MutT protein can hydrolyze an oxidized dGTP nucleotide, 8-oxo-deoxyguanosine triphosphate (8-oxo-dGTP), back to the monophosphate form 8-oxo-dGMP (14). 8-oxo-dGTP is a potent mutagenic nucleotide that is readily incorporated into DNA opposite template C or A (14), with a preference for template A (15,16). Therefore, by cleaning the 8-oxo-dGTP pool, MutT reduces spontaneous transversion mutations ∼1000-fold (12–13,17). In addition, MutT can also act on 8-oxo-guanosine triphosphate (8-oxo-GTP) and convert it to 8-oxo-GMP averting mutagenic nucleotide incorporation into RNA (18). *mutT* mutants are known to exhibit phenotypes independent of DNA mutational effects, which have been attributed to a decrease in transcription fidelity (18–20). The persistence of oxidized ribonucleotides in the available nucleotide pool and the subsequent incorporation of 8-oxo-GTP into mRNA, causing T to G transversions in the nascent transcript, was assumed to be the mechanism for RNA errors and these phenotypes. It was suggested that the absence of MutT can increase the readthrough of a stop codon mutation through 8-oxo-GTP incorporation generating a 30-fold increase in functional protein levels in *mutT* cultures compared to wild-type cultures (18). Such RNA infidelity in *mutT* strains may account for the accumulation of misfolded proteins (19) and the observed cytotoxicity of aminoglycoside antibiotics (20).

To investigate the role of MutT on transcription fidelity, we have used our bistable *lac* assay that is sensitive to transcription errors and find no increase in epigenetic switch frequency in the absence of MutT function; we also show that the phenotype previously attributed to transcription errors [partial phenotypic suppression or leakiness (18)] is principally due to mutagenic events in the DNA of the cells and not due to the incorporation of oxidized GTP into mRNA. In light of our results we suggest that other observed *mutT* phenotypes, such as protein mistranslation or antibiotic sensitivity, that have been attributed to 8-oxo-GTP require other explanations than simply 8-oxo-GTP misincorporation into mRNA.

## MATERIALS AND METHODS

### Bacterial strains

All strains used in this study are derived from the wild-type sequenced *E. coli* MG1655 strain or strain CC101 (gift of Susan Rosenberg, Baylor College of Medicine, USA) and are found in Table 1. Manipulation of the MG1655 and CC101 genomes was accomplished by standard methodologies (21,22). The *dnaE941* allele (gift of Roel Schaaper, National Institute of Environmental Health Sciences, USA) and the Δ*mutT* and Δ*mutY* deletion mutations (Keio Collection, Keio University, Japan) (23) were moved into CC101 by P1 transduction; the *lacI*^q^ allele (Coli Genetic Stock Center, Yale, USA) was moved into CH1118 by P1 transduction. The kanamycin resistance cassettes were removed by a pCP20 flippase reaction.

**Table 1.** Bacterial strains

Strain	Genotype	Reference
CH30	MG1655: *λ^−^*, *rph-1*	laboratory stock
CH458	MG1655 *lacZYA::gfp-cat^R^*	(5)
CH5201	*ΔlacI::cmR lacZYA::gfp_FRT_* (CH2163 with pKD46 lost)	(6)
CH1071	NCM514 *lacIp-4000* (*lacI*^q^) *zah-2224::catR λ^−^*, *rph-1*	CGSC 8249
CH1118	MG1655 *lacZYA::gfp_FRT_*	(5)
CH1143	MG1655 *lacI*^q^*zah-2224::cat^R^ lacZYA::gfp_FRT_*	CH1118 x P1 (CH1071)
BW25113	F^−^, *Δ(araD-araB)567*, *ΔlacZ4787(::rrnB-3)*, *λ^−^*, *rph-1*, *Δ(rhaD-rhaB)568*, *hsdR514*	(23)
CH584	BW25113 *mutY::kn^R^*	JW2928 (23)
CH336	BW25113 *mutT::kn^R^*	JW0097 (23)
CH4198	NR10778: *ara-9, fhuA1, lacY1 or lacZ4, tsx-3, supE44, galK2*, λ^−^, *hisG4(oc), rfbD1?, trp-3(Oc), rpsL8 or rpsL9 malA1* (λ^r^), *metE46, mtl-1, thi-1, dnaE941, zae502::Tn10, lacZ118(oc)*	(37)
CH353	MG1655 *mutT::kn^R^*	CH30 x P1 (CH336)
CH605	MG1655 *mutY::kn^R^*	CH30 x P1 (CH584)
CH586	CC101: F` *lacI^−^Z^−^ proA^+^B^+^; ara* Δ*(gpt-lac)5*	(17) via S.M.Rosenberg
CH2956	F` *lacI^−^Z^+^ proA^+^B^+^; ara* Δ*(gpt-lac)5*	*lacZ*^+^ revertant
CH952	CH586 *mutT::kn*^R^	CH586 x P1 (CH353)
CH4404	CH586 Δ*mutT_FRT_*	CH952 flipped
CH4406	CH586 Δ*mutT_FRT_ mutY::kn^R^*	CH4404 x P1 (CH605)
CH4408	CH586 Δ*mutT_FRT_* Δ*mutY_FRT_*	CH4406 flipped
CH4412	CH586 Δ*mutT_FRT_* Δ*mutY_FRT_ dnaE941 zae502::Tn10*	CH4408 x P1 (CH4198)
CH4416	CH586 Δ*mutT_FRT_ dnaE941 zae502::Tn10*	CH4404 x P1 (CH4198)
CH458	MG1655 *lacZYA*::*gfp-cm^R^*	(5)
CH505	MG1655 *lacZYA*::*gfp-cmR mutT::kn^R^*	CH458 x P1 (CH353)

### Growth conditions and media

To determine the level of Lac^+^ revertants for each strain, a bacterial culture grown in LB media, was diluted and ∼200 cells were seeded to new tubes containing fresh LB media and shaken at 37°C overnight. The cultures were then washed 3× in minimal A salts (21) and then aliquots were plated onto minimal A lactose plates for Lac^+^ revertant determination and onto minimal A glucose plates for viability. Mutation frequency was determined by dividing Lac^+^ revertants by the number of viable cells in the culture (17,21). To determine the *lacI*^+^ to *lacI*^−^ genetic mutation frequency, a bacterial culture grown in minimal A succinate media, was diluted and ∼200 cells were seeded to new tubes containing fresh minimal A succinate media and shaken at 37°C overnight. The cultures were then washed 3× in minimal A salts and we selected for colony forming ability on agar plates containing phenyl-β-D-galactoside (Pgal; 75 μg/ml) as the sole carbon source, and onto minimal A glucose plates for viability. Only cells constitutively expressing β-galactosidase (*lacI^−^* and *lacO*^c^ mutants) can form colonies on Pgal plates. Mutation frequency was determined by dividing the number of *lacI*^−^ mutants by the number of viable cells in the culture.

To demonstrate hysteresis and bistability in *lac* operon expression in single cells, a bacterial culture grown in minimal A salts plus MgSO_4_ (1 mM) with succinate (0.2%), was diluted 1:5 in fresh medium with (ON culture) or without 1-mM TMG (OFF culture) and shaken at 37°C for 7 h. After this induction period, the two cultures were individually diluted and ∼200 cells were seeded to new tubes containing fresh medium that contained varying amounts of TMG, and shaken at 37°C for 42 h. Flow cytometry was used to determine the percentage of cells that were induced for *lac* operon expression (ON cells), as previously described (6).

To determine epigenetic switch frequencies, a bacterial culture grown in minimal succinate media, was diluted and ∼200 cells were seeded to new tubes containing fresh medium, with a maintenance level of 6-μM TMG, and shaken at 37°C for 42 h, as previously described (6), and subjected to flow cytometry.

A reconstruction test was performed to determine the dynamic range of the β-galactosidase assay. Overnight LB cultures of CH586 (*lacZ*^−^) and a Lac^+^ revertant strain (CH2956) were used; the CH2956 culture was 10-fold serially diluted into CH586 to make 1 ml in total cell volume, in duplicate, and with these reconstructed Z^+^:Z^−^ populations β-galactosidase assays were performed. The cell titers of the initial cultures were determined by diluting and plating onto LB plates; the number of Lac^+^ revertants in the initial CH586 culture was determined by plating onto minimal lactose plates, and diluting and plating onto minimal glucose plates for cell titer.

### Single-molecule mRNA fluorescent *in situ* hybridization (smFISH)

The smFISH protocol has been described in detail (24,25). Fluorescently labeled oligonucleotide probe sequences designed against the *lacI* transcript (purchased from Biosearch Technologies, USA) are described in Supplementary Table S1. Bacterial strains for smFISH analysis were grown in minimal A salts with succinate (0.2%) and thiamine at 37°C. The estimation of mRNA number in the cell relies on quantifying localized fluorescence and is not achieved by counting discrete spots. The number of bound probes is measured on the basis of the total fluorescence intensity (photon flux) of the spots, without requiring that individual mRNAs appear as separate spots. By performing a calibration step, the total intensity of spots in the cell can then be converted to the number of target mRNAs (25).

### Flow cytometry

To determine the percentage of cells that were induced for *lac* operon expression (ON cells), 10 μl of culture was diluted into 300 μl filtered minimal A salts plus MgSO_4_ (1 mM) and subjected to flow cytometry analysis with GFP fluorescence measured in a BD FACSCanto II Flow Cytometer (Becton, Dickinson and Company, USA) with Diva acquisition software (Becton Dickinson) and FloJo analysis software (Tree Star, Inc. USA). To monitor fluorescent cells in a culture we used a narrow gating for forward and side scattering so that the most represented cell population was evaluated. For each independent culture 10 000 cells were interrogated, as previously described (6).

### β-galactosidase levels monitored over time by an automated microplate reader

Overnight LB cultures of each strain were diluted and ∼200 cells were seeded into fresh LB media containing 0.5 mg/ml 5-bromo-4-chloro-3-indolyl-β-D-galactoside (Xgal) in a microtiter dish (200 μl media per well) and grown at 37°C with shaking in a BioTek Synergy™ 2 multi-detection automated microplate reader. Readings were recorded every hour for 46 h (OD_600_ to monitor cell growth; OD_615_ to monitor Xgal cleavage). OD_600_ readings from the LB control wells (cells but no Xgal) were subtracted from OD_615_ readings from wells containing the same strain with Xgal to normalize readings to account for cell growth.

### β-galactosidase assay

Cells were grown in LB media and β-galactosidase levels were determined by the method of Miller (21). A Z buffer ortho-nitrophenyl-β-D-galactopyranoside (ONPG) control β-galactosidase assay (no cells) was included due to the prolonged time of some of the reactions with very low enzyme levels.

## RESULTS AND DISCUSSION

### Stochastic switching in the *lac* bistable gene network in Δ*mutT* cells

**Figure 1.**
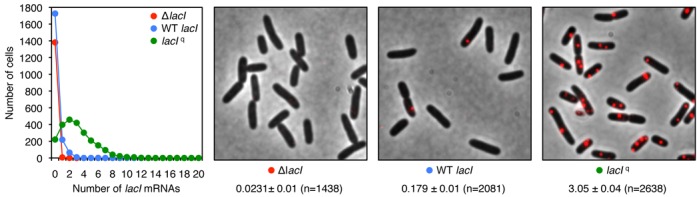
Single-molecule FISH (smFISH) to characterize *lacI* mRNA copy number statistics. Typical images of smFISH-labeled cells in different strain backgrounds (Δ*lacI*, CH5201; wild-type *lacI*, CH458; *lacI*^q^, CH1143). An overlay of the phase contrast (grayscale) and smFISH probes targeting the *lacI* gene (red) is shown. *n*, number of cells monitored. Mean and standard error values for absolute *lacI* mRNA numbers per cell are shown for each strain. The histogram shows the distribution of *lacI* mRNA molecules per cell observed within each strain population monitored.

Although it is clear that incorporation of 8-oxo-dGTP into DNA can have mutagenic consequences for the cell, with heritable phenotypic consequences, it remains unknown if incorporation of 8-oxo-GTP into RNA can also have heritable phenotypic consequences for the cell (26). To study the effect of *mutT* on heritable phenotypic change, we used the bistable *lac* switch assay in *E. coli*. This bistable switch assay is sensitive to transcription errors: we previously observed that a 5-fold decrease in transcription fidelity due to an RNA polymerase mutation [measured both *in vitro* and *in vivo* (27)] leads to a 4- to 6-fold increase in epigenetic switching frequency in our bistable *lac* assay (5). Moreover, the number of *lacI* mRNA per cell is very low making this system susceptible to transcription errors. Based on equilibrium dialysis against radioactive isopropyl-thio-galactoside it was estimated that there are about five *lac* repressor tetramers per cell (10). It was therefore suggested that the *lacI* gene has an inefficient promoter and that only one or two mRNA molecules are synthesized per *lacI* gene per generation (28). We directly measured the number of *lacI* mRNA molecules in single cells using single-molecule mRNA fluorescent *in situ* hybridization (smFISH) (24,25) to quantify mRNA statistics in three *E. coli* strains: an entire deletion of the *lacI* gene (CH5201), the wild-type *lacI* promoter (CH458) and the *lacI* up-promoter *I*^q^ (CH1143; Figure 1). Fully 86% of wild-type *lacI* cells do not exhibit any *lacI* mRNA, 11% of cells have one *lacI* mRNA and 3% have two or more *lacI* mRNA per cell at any given time (2081 cells monitored); see Figure 1. No *lacI* mRNA molecules were observed in cells that have the entire *lacI* gene deleted. A mutant that makes about 10× more *lac* repressor, *lacI*^q^, has been previously isolated (28) which carries an up-mutation at the −35 position of the *lacI* promoter (29). smFISH analysis showed that the *lacI*^q^ strain exhibited an average of 3.05 *lacI* mRNA per cell (2638 cells monitored; Figure 1). This is the first demonstration of wild-type *lacI* mRNA statistics and is in excellent agreement with the low *lac* repressor numbers indirectly determined during the initial isolation of the *lac* repressor almost 50 years ago (10). Thus, these results suggest that wild-type *lac* repressor production is subject to large fluctuations in protein number, due to rare stochastic transcription events (30), making the bistable switch system sensitive to transcription errors during *lacI* mRNA production (31).

**Figure 2.**
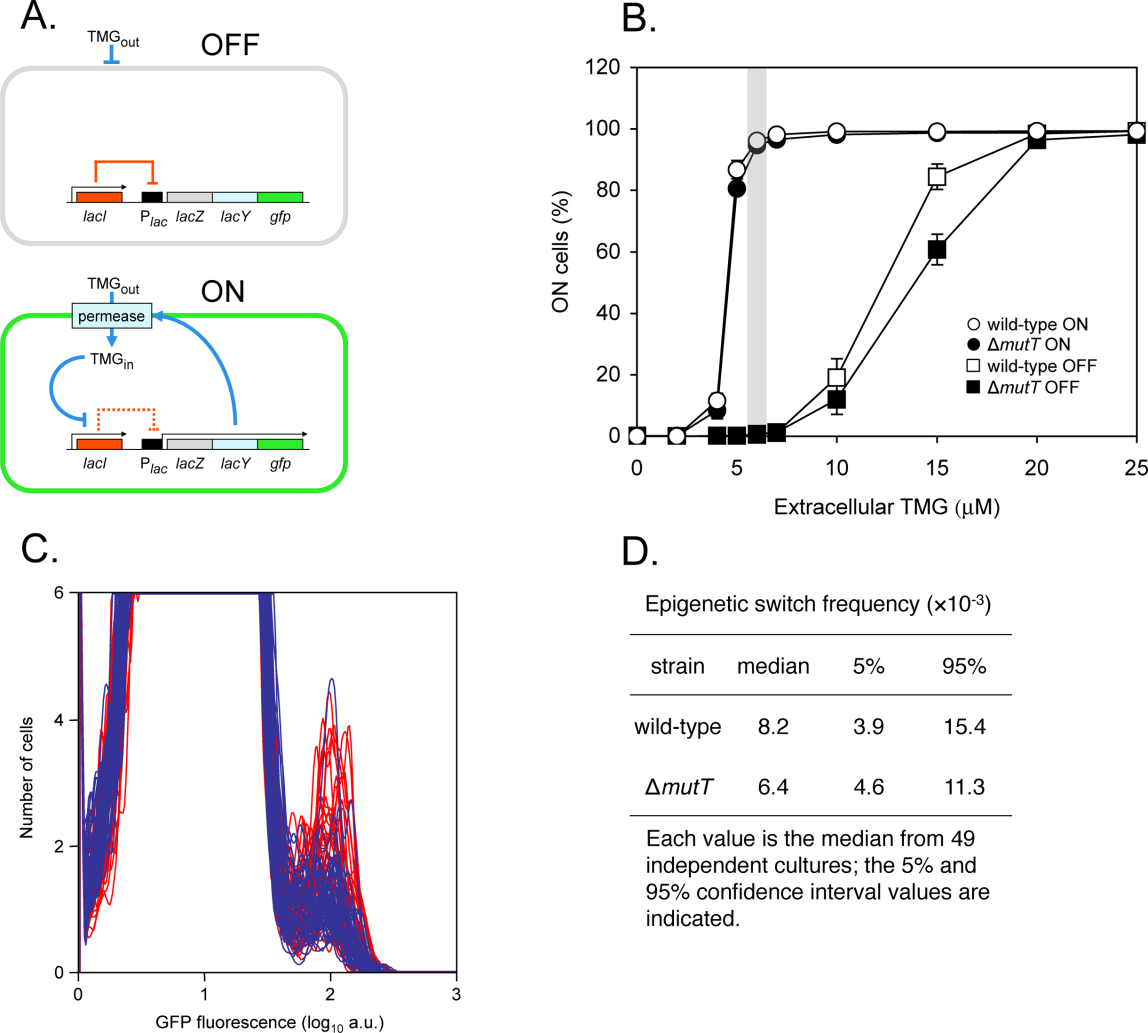
Stochastic switching in the *lac* bistable gene network. (**A**) Under maintenance conditions, the *lac* operon is OFF when the *lac* repressor is bound to the *lac* operator (indicated by the solid red line) and the inducer thio-methylgalactoside (TMG) remains extracellular; stochastic events that lead to a transient derepression of the *lac* operon will result in a burst of *lac* operon functions and the appearance of permease will initiate an autocatalytic positive-feedback response (indicated by solid blue lines), which will heritably maintain the ON state (TMG induces an allosteric transition in *lac* repressor, indicated by the dashed red line, so that it no longer binds to the *lac* operator), and the cell will exhibit green fluorescence. (**B**) Wild-type (CH458) and Δ*mutT* (CH505) cells that were originally ON or OFF were sub-cultured and grown in media containing various concentrations of TMG. Each value is the average ±SD from four independent cultures. The shaded area highlights the maintenance concentration of 6-μM TMG for these strains. (**C**) OFF wild-type cells (red histograms) and OFF Δ*mutT* cells (blue histograms) were diluted and grown in media containing 6-μM TMG. After 42 h growth, flow cytometry was performed to determine the frequency of epigenetically ON cells in 49 independent cultures of each strain; the Δ*mutT* histograms are superimposed over the wild-type histograms (10^4^ cells interrogated for each histogram). (**D**) The Δ*mutT* epigenetic-switch frequency is not significantly increased over the wild-type value (Mann–Whitney Rank Sum Test, *P* = 0.13).

To determine the proportion of cells that switch to the ON state for *lac* operon expression, we used a *lacZYA::gfp* construct expressing β-galactosidase, galactoside permease and green fluorescent protein (5). During growth of OFF cells in a maintenance concentration of TMG (6-μM TMG; see Figure 2A and B), if a cell suffers a stochastic event leading to derepression of the *lac* operon, this transient derepression will trigger permease synthesis and activation of the autocatalytic positive-feedback loop, resulting in green fluorescent cells (5,6). As a result, the OFF state will transition to the ON state and be heritably maintained in the following generations, mimicking *lacI* mutation in this system (Figure 2A). To determine the epigenetic switch frequency, we measured the number of green cells within the resulting cultures by flow cytometry (Figure 2C). We calculate the epigenetic switch frequency as number of ON cells over the total number of cells interrogated, following the convention used in determining *lacI*^−^ mutation frequencies in a population (21). The observed ON switch frequency is therefore dependent on both the number of switch events that have occurred and the number of generations after a discrete switch event has occurred, as in a classical fluctuation test (see ‘Materials and Methods’ section).

When we measured the epigenetic switch frequency in our bistable switch assay in a Δ*mutT* strain (CH505), we did not observe any increase in epigenetic switch frequency compared to wild-type cells (Mann–Whitney Rank Sum Test, *P* = 0.13) (Figure 2D). Both wild-type (CH458) and *mutT* strains exhibit bistability and similar hysteresis patterns, and the maintenance concentration of 6-μM TMG is the same for both strains (Figure 2B). As expected, we observed a 130-fold induction in *lacI*^+^ to *lacI*^−^ forward mutation frequency in the *mutT* strain over the *mutT*^+^ strain (3.1 × 10^−4^ ± 4.1 × 10^−4^ SD for the *mutT* strain, three independent experiments; 2.4 × 10^−6^ ± 1.5 × 10^−6^ SD for the wild-type strain, nine independent experiments), which is similar to mutation induction found in other studies of *mutT* mutagenesis in the *lacI* system (32).

Our system directly compares the frequency of permanent and transient errors in information transfer that lead to the same phenotype (*lac* operon ON) in the same system (5). While the genetic mutation frequency is increased in Δ*mutT* cells, there is no observed increase in the epigenetic switch frequency compared to *mutT*^+^ cells. This result is intriguing because the susceptible sites of *mutT*-mediated mutation in the *lacI* gene should be equally susceptible to *mutT*-mediated RNA error in *lacI* mRNA generating non-functional repressor. Indeed, throughout the *lacI* gene there are 23 sites where 8-oxo-dGTP/8-oxo-GTP incorporated opposite A on the transcribed strand would give a non-functional *lac* repressor, whereas there are 14 sites where 8-oxo-dGTP incorporated opposite A on the non-transcribed strand would give a non-functional *lac* repressor (32–34). Therefore, the *lacI* transcript is a robust target to monitor the phenotypic consequences of 8-oxo-GTP incorporation opposite A residues during transcription.

### Reducing A:T → C:G DNA mutation in a Δ*mutT* strain

The putative role for MutT in transcription fidelity was first observed using a *lacZ* amber mutant strain specific to *mutT*-mediated mutation (18). We note that study used the F′ from CC101 (carrying the *lacZ*_amber_ mutation: F′ *lacI^−^Z^−^ proA^+^B^+^*) in a *ara* Δ*(gpt-lac)5 thi trpE9777* background; we use the original CC101 strain (F′ *lacI^−^Z^−^ proA^+^B^+^; ara* Δ*(gpt-lac)5*; Table 1) and our results should be directly comparable (both strains that carry the CC101 F′ will be referred to as CC101). *E. coli* strain CC101 (CH586) measures reversion from Lac^−^ to Lac^+^ specifically via the A:T → C:G transversion at codon 461 of the *lacZ* gene; at this site the wild-type GAG Glu codon is now a TAG amber nonsense mutation (17). At the DNA level, in a *mutT* strain such A:T → C:G transversions are specifically produced through 8-oxo-dGTP incorporation opposite the initial A of the amber nonsense codon on the transcribed DNA strand during replication (Supplementary Figure S1). Similarly, at the RNA level, partial phenotypic suppression (leakiness) may occur through 8-oxo-GTP incorporation opposite the same A of the amber nonsense codon on the transcribed DNA strand during mRNA transcription (Supplementary Figure S1). As a result of 8-oxo-GTP incorporation, the ^3′^ATC^5′^ nonsense codon will be transcribed as ^5′^G*AG^3′^ (G* indicates 8-oxoG) in the nascent transcript and translated as a Glu residue by tRNA with a ^3′^CUU^5′^ anticodon sequence (35), providing functional β-galactosidase. Therefore, both permanent DNA mutations and transient mRNA misincorporations at this nucleotide position may contribute to the β-galactosidase levels observed in *mutT* cultures.

We sought to diminish the mutational burden of the absence of MutT function in the CC101 strain to allow the phenotypic consequences of 8-oxo-GTP incorporation into RNA to be enhanced. It has been shown that the absence of MutY function can decrease A:T → C:G mutations in a CC101 Δ*mutT* strain, i.e. MutY function is mutagenic in the absence of MutT function in *E. coli* (16,36). MutY is an adenine glycosylase that removes the A from A:G (and A:G*) mismatches with no apparent strand specificity: MutY does not distinguish between an A that is incorrectly incorporated opposite a template G (or G*) during DNA replication, nor a template A paired with a misincorporated G (or G*) (see Supplementary Figure S2). Vidmar and Cupples (36) observed a 71% decrease in A:T → C:G mutants in a CC101 *mutT mutY* strain compared to a CC101 *mutT* strain; we also find a similar 70% decrease in A:T → C:G mutants in an independently created CC101 *mutT mutY* strain (CH4406) compared to a *mutT* strain (CH4404) (Table 2).

**Table 2.** Reducing A:T → C:G mutation in a Δ*mutT* strain

Strain	Lac^+^ revertants per 10^8^ cells	Reduction in Δ*mutT* consequences
CC101 *mutT^+^*	0.2 ± 0.2	
CC101 Δ*mutT*	1184 ± 313	
CC101 Δ*mutT dnaE941*	476 ± 233	60%
CC101 Δ*mutT* Δ*mutY*	352 ± 27	70%
CC101 Δ*mutT* Δ*mutY dnaE941*	153 ± 20	87%

Frequencies are means (±SD) for eight independent cultures per strain.

To further decrease the mutational burden of the absence of MutT function in the CC101 strain, we replaced the wild-type *dnaE* gene with the *dnaE941* anti-mutator allele. The *dnaE941* allele encodes a DNA polymerase that was isolated as a suppressor of the high mutability of a *mutT* mutator strain (37). The *dnaE941* allele in a *mutT* strain decreased the level of mutations to rifampicin resistance by 60% compared to a *dnaE*^+^ *mutT* strain (37). We also observe a 60% decrease in A:T → C:G events in the CC101 *mutT dnaE941* strain (CH4416) compared to the CC101 *mutT* strain (Table 2). Although the mechanism by which the *dnaE941* anti-mutator increases the fidelity of DNA replication is not known, it has been suggested to be due to an increase in polymerase base selectivity or an increase in exonucleolytic proofreading ability (37).

Moreover, when we combine the *dnaE941* antimutator allele with an absence of MutY function we observe an 87% decrease in A:T → C:G events (strain CC101 *mutT mutY dnaE941* (CH4412) versus strain CC101 *mutT*; Table 2). Therefore, the phenotypic consequences of transient errors in RNA transcription can now be assessed with less of the confounding influence of permanent Lac^+^ mutants in the bacterial culture. We emphasize that the levels of 8-oxo-dGTP and 8-oxo-GTP will be unchanged in all the *mutT* strains, but the DNA mutational consequences of those same levels of oxidized nucleotide pools will be different (diminished) in the *mutT* strains that carry the additional mutated or altered *mutY* and *dnaE* alleles. We note that cultures of the CC101 Δ*mutT* Δ*mutY dnaE941* strain still generate over 500-fold more Lac^+^ mutants than the wild-type CC101 strain (Table 2).

### Lac^+^ revertants can account for increased β-galactosidase levels in Δ*mutT* cultures

**Figure 3.**
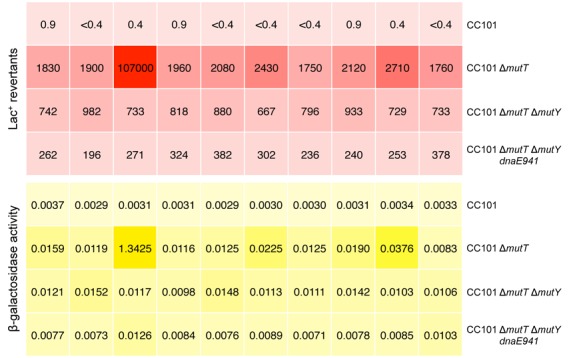
Lac^+^ mutation frequencies and β-galactosidase enzyme levels in CC101 and Δ*mutT* compromised cultures. The upper red heat map describes Lac^+^ revertants per 10^8^ cells with each panel representing an independent culture (with actual revertant numbers presented); the lower yellow heat map describes β-galactosidase enzyme levels with each panel representing the same independent culture found in the corresponding position in the red heat map (actual activities in Miller units are presented). Each row depicts independent cultures of the same strain.

To concurrently study the genotypic and phenotypic effect of the absence of MutT function, we measured mutation frequency and β-galactosidase activity from the same culture in wild-type CC101, Δ*mutT* and the different strain backgrounds that exhibit reduced mutational load for *mutT-*mediated mutagenesis. Figure 3 shows the Lac^+^ reversion mutation frequencies and β-galactosidase enzyme activities for 10 independent CC101 and Δ*mutT* compromised cultures in the form of heat maps, with each panel representing an independent culture; the corresponding panels in the two maps represent the two methods of analysis undertaken on the same culture. When the mutational jackpot (row 2, column 3) is omitted from analysis, the same trend is observed as found in Table 2: the mutational consequences of the Δ*mutT* allele is decreased by 61% in a CC101 Δ*mutT* Δ*mutY* strain, and 86% in a CC101 Δ*mutT* Δ*mutY dnaE941* strain in this independent experiment (Supplementary Table S2). Again, omitting the jackpot culture, we observe a 5.3-, 3.8-, 2.7-fold increase in β-galactosidase enzyme levels for the Δ*mutT*, Δ*mutT* Δ*mutY* and Δ*mutT* Δ*mutY dnaE941* strains, respectively, over that observed in the wild-type CC101 strain (Supplementary Table S2). We observe that cultures of the Δ*mutT* Δ*mutY dnaE941* strain generate over 500-fold more Lac^+^ mutants than the wild-type CC101 strain, but exhibit only a 2.7-fold increase in β-galactosidase enzyme level in the culture.

**Figure 4.**
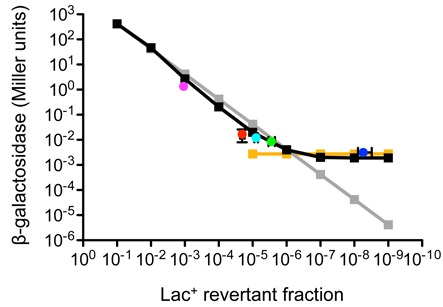
A reconstruction test of the contribution of Lac^+^ revertants within a Lac^−^ population monitored by β-galactosidase assay. Black squares represent 10-fold serial dilutions of Z^+^ into Z^−^ cells and then β-galactosidase assays were performed. Yellow squares represent the Z buffer ONPG (no cells) control assayed at the same time as the 1:10^5^ to 1:10^9^ (Z^+^:Z^−^) dilution time points. Gray squares represent the theoretical decrease in β-galactosidase units if the initial time point (1:10 dilution) β-galactosidase level is maintained throughout dilution in the reconstruction experiment. CC101 (dark blue circle), CC101 Δ*mutT* jackpot (purple circle), CC101 Δ*mutT* (red circle), CC101 Δ*mutT* Δ*mutY* (light blue circle), CC101 Δ*mutT* Δ*mutY dnaE941* (green circle) values are from Figure 3 and Table 3; mean ± SD for 9 (red) or 10 (light blue, green, dark blue) cultures (the purple jackpot is a singular culture).

Our result is in contrast to that of Taddei *et al.* (18) not only in the fold increase in β-galactosidase levels, they observed a 30-fold increase in β-galactosidase levels ( leakiness ) in CC101 Δ*mutT* cultures compared to CC101 cultures, but also in the interpretation of the fold increase in β-galactosidase levels observed in Δ*mutT* cultures. The increased CC101 Δ*mutT* leakiness in that study was attributed to errors in transcription, with 8-oxo-GTP being incorporated into the nascent transcript across from the template A of the ^3′^ATC^5′^ nonsense mutation (Supplementary Figure S1); the possibility that this increased β-galactosidase enzyme level in Δ*mutT* cultures was due to Lac^+^ revertant mutations arising in the growing population, i.e. 8-oxo-dGTP being incorporated opposite the template A during DNA replication, was discounted (18). The following formula was previously used to consider the contribution of Lac^+^ revertants to the β-galactosidase activity of the population, *M*(pop), measured in Miller units: *M*(pop) = *M*(lac^+^)*f*(lac^+^) + *M*(lac^−^)*f*(lac^−^) where *M*(lac^+^) and *M*(lac^−^) are β-galactosidase activities of Lac^+^ and Lac^−^ cells, and *f*(lac^+^) and *f*(lac^−^) their respective frequencies (18). Therefore, for an overnight CC101 culture: the frequency of Lac^−^ is close to 1 and the frequency of A:T → C:G Lac^+^ revertants is ∼10^−5^ in the Δ*mutT* context (however, this value will fluctuate due to the stochastic nature of mutation appearance during growth of the bacterial culture). The wild-type level of β-galactosidase activity in a fully induced Lac^+^ CC101 culture is ∼3800 Miller units (38); we observe the same enzyme levels in fully induced *lacZ*^+^ cultures (Figure 4; data not shown). It was therefore argued (18) that the increase in β-galactosidase concentration Taddei *et al.* observed in a *mutT* strain was not due to reversion mutations [*M*(pop) = 0.38; *f*(lac^+^) = 10^−5^; *M*(lac^+^) = 38 000], because each revertant would need to produce 10-fold *more* β-galactosidase Miller units than the value obtained for a fully induced wild-type *lacZ* gene.

Here, we used the same formula and found that the β-galactosidase activities in the CC101 Δ*mutT* background, and in all the backgrounds that reduce the mutational burden of the absence of MutT function, can be readily explained solely by the generation of Lac^+^ DNA revertant bacteria that arise during the growth of the cultures. All the revertant Lac^+^ β-galactosidase levels observed in the Δ*mutT* strain, and the Δ*mutT* derivatives, approach the β-galactosidase levels made by a fully induced wild-type *lacZ* gene, or are *less* than the β-galactosidase levels made in a fully induced wild-type *lacZ* gene, including the Δ*mutT* mutation jackpot culture down to the Δ*mutT* Δ*mutY dnaE941* background, which is a ∼400-fold difference in Lac^+^ mutation frequency (Table 3). In the analysis of Taddei *et al.*, bacterial cultures containing more than 10^−5^ Lac^+^ revertants were said to be discarded from their analysis (18); here, we do not discard any results but retain them for their information content. By performing both mutation assays and β-galactosidase assays for each culture, which in total forms a robust fluctuation distribution, we show that Lac^+^ revertants can completely account for the increase in β-galactosidase levels in all these cultures even though the reversion frequencies from CC101 Δ*mutT* cultures to CC101 Δ*mutT* Δ*mutY dnaE941* cultures vary from 10^−3^ to 10^−6^ (Figure 3; Table 3).

**Table 3.** Contribution of DNA errors to the observed population β-galactosidase levels

Strain	*M*(pop)	=	*M*(lac^+^)	*f*(lac^+^)	+	*M*(lac^−^)	*f*(lac^−^)
Wild-type Lac^+^	3800		3800	1		ND	∼10^−6^
CC101 Δ*mutT*	0.0169		805	2.1 × 10^−5^		ND	1
CC101 Δ*mutT* jackpot	1.3425		1220	1.1 × 10^−3^		ND	1
CC101 Δ*mutT* Δ*mutY*	0.0121		1513	8 × 10^−6^		ND	1
CC101 Δ*mutT* Δ*mutY dnaE941*	0.00862		3079	2.8 × 10^−6^		ND	1

*f*(lac^+^) values are from Figure 3 and Supplementary Table S2. *M*(lac^+^) is simply *M*(pop) divided by *f*(lac^+^). *M*(lac^−^) was not determined (ND). The CC101 Δ*mutT* jackpot culture is observed in row 2, column 3 in Figure 3.

When we plot these results (Figure 3; Table 3) against the reconstruction experiment we generated the situation becomes clear (Figure 4). A LacZ^+^ revertant culture (CH2956) was 10-fold serially diluted into a CC101 LacZ^−^ culture (the initial LB titers of the two cultures were the same, 4.4 × 10^9^ cells per ml; the number of Lac^+^ revertants in the initial CC101 culture was 0.2 per 10^8^ cells, and therefore this low Lac^+^ number renders any contribution to the total β-galactosidase units in the reconstruction test negligible) and β-galactosidase assays were performed. As the number of Lac^+^ revertants falls, the β-galactosidase units in the reconstruction test falls in parallel until the level of 1 Z^+^: 10^6^ Z^−^ is achieved; after that point, any further dilution in Lac^+^ numbers does not affect the observed β-galactosidase units level which levels out at the same β-galactosidase unit level as the Z buffer ONPG control. The numbers of Lac^+^ revertants, and the corresponding β-galactosidase units, for CC101, Δ*mutT* and all other the *mutT*-compromised strains, including the Δ*mutT* jackpot culture, all fall on, or close to, the reconstruction line (see Figure 4). It is clear that the dynamic range of the β-galactosidase assay has been exceeded when the CC101 strain is monitored: in theory, the CC101 strain should exhibit a value more than 100-fold less than that observed (the CC101 level is simply the level of the Z buffer ONPG control); in practice, the β-galactosidase level in CC101 is over-estimated (by at least 100-fold), and therefore the fold difference of the Δ*mutT*, and all the other *mutT*-compromised strains, over the CC101 level is under-estimated. We note that our CC101 Δ*mutT* Δ*mutY dnaE941* cultures contain over 500-fold more Lac^+^ revertants than the wild-type CC101 strain, and although these CC101 Δ*mutT* Δ*mutY dnaE941* cultures exhibit β-galactosidase levels that can be completely accounted for by Lac^+^ revertants [*M*(lac^+^) is 3079 Miller units; see Table 3], they exhibit only a 2.7-fold increase in β-galactosidase units over CC101. Figure 4 provides the answer to this conundrum, namely, that the CC101 Δ*mutT* Δ*mutY dnaE941* strain is being effectively assayed for β-galactosidase content in the culture, while the CC101 strain is not being effectively assayed for β-galactosidase content in the culture, and the correction for this mis-monitoring would be over 100-fold.

We demonstrate that our observed β-galactosidase levels in CC101 Δ*mutT* cultures can be accounted for by the Lac^+^ revertant numbers we found in the same cultures, unlike the initial study, where the β-galactosidase levels observed were stated to be too high to be accounted for by Lac^+^ revertant numbers (18). It is difficult to address this discrepancy, but our reconstruction test results would suggest that the initial study may have contained more Lac^+^ revertants than appreciated in the Δ*mutT* cultures, and the authors did not realize that the purported CC101 β-galactosidase readings are beyond the range of the β-galactosidase assay (Figure 4). We do note that in a recent study (35), it was found that using a similar system that also monitored partial phenotypic suppression using a different *lacZ* amber mutation, a 1.5-fold increase in leakiness was observed in Δ*mutT* cultures compared to wild-type cultures, a result more in line with what we observe here (5.3- to 2.7-fold), and not what was previously observed (∼30-fold) in the CC101 background (18,39). This discrepancy in *lacZ_amber_* leakiness (1.5- versus the 30-fold increase observed before at codon 461 of *lacZ* in a Δ*mutT* background) was not addressed by Inokuchi *et al.* (35). The system of Inokuchi *et al.* utilized a single base substitution in the glutamine codon (CAG) at the position 1456 of the *lacZ* gene, creating an amber nonsense mutation at codon 486; incorporation of 8-oxo-GTP opposite the A of the nonsense codon on the transcribed DNA strand would allow the incorporation of a glutamic acid residue at this site that will also provide essentially the same β-galactosidase activity as the wild-type enzyme (35). Therefore, we did not find an unaccountable increase in β-galactosidase levels in a Δ*mutT* strain at site 461 in *lacZ_amber_* as found previously (18,39), and Inokuchi *et al.* did not find any significant increase at another site, 486 in *lacZ*, in a system that was created to specifically assess leakiness due to 8-oxo-GTP incorporation in the *lacZ* transcript (35). As was noted before (26), other *trpE* and *tyrA* auxotrophs with ochre mutations do not demonstrate any leakiness in a Δ*mutT* background (40).

Finally, we note that the high spontaneous level of A:T → C:G mutations in *mutT* strains is completely suppressed when the *mutT* cells are cultured in anaerobic conditions, indicating the essential role of oxygen in *mutT*-mediated mutagenesis (41–43). It was also observed that anaerobic conditions reduced transcriptional leakiness in a *mutT* strain almost to the wild-type level, consistent with the involvement of reactive oxygen species (18). It has been previously argued (18) that mutation and leakiness in *mutT* cultures exhibited differential responses during anaerobiosis (mutation was not reduced; leakiness was reduced) and therefore this would be consistent with the idea that *mutT*-mediated mutations are not responsible for the observed increase in β-galactosidase levels in a *mutT* aerobic culture; in light of other studies (41–43) this argument is no longer tenable.

### Fluctuation analysis of β-galactosidase appearance

**Figure 5.**
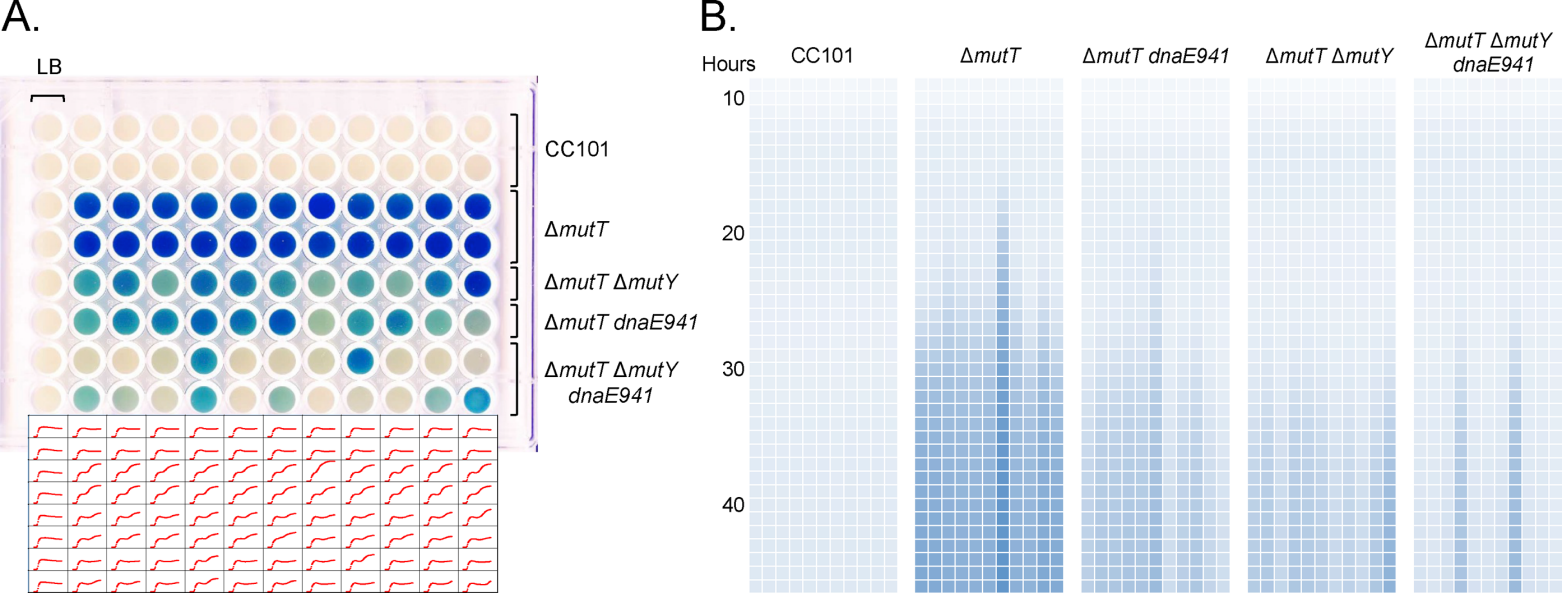
A fluctuation test analysis of Lac^+^ revertants in CC101 and Δ*mutT* compromised strains. Overnight LB cultures of each strain were diluted and ∼200 cells were seeded into fresh LB media containing 0.5 mg/ml Xgal in a microtiter dish (200 μl per well) and grown at 37°C with shaking in an automated plate reader. Readings were recorded every hour for 46 h (OD_600_ to monitor cell growth; OD_615_ to monitor Xgal cleavage). (**A**) False color microtiter plate after 46 h growth. Rows 1 and 2, CC101; rows 2 and 3, CC101 Δ*mutT*; row 5, CC101 Δ*mutT* Δ*mutY*; row 6, CC101 Δ*mutT dnaE941*; rows 7 and 8, CC101 Δ*mutT* Δ*mutY dnaE941.* The first column is an LB control (cells but no Xgal; OD_600_ readings from these wells were subtracted from OD_615_ readings from wells containing the same strain with Xgal to normalize readings to account for cell growth as shown in (**B**)); all other wells contain LB plus Xgal. The corresponding OD_615_ traces are found beneath the microtiter plate. (B) Heat map representation of the OD_615_ scans over time showing the fluctuation nature of the observance of Lac^+^ mutation. Each row represents normalized hourly OD_615_ readings starting at hour 9 and ending at hour 46. First panel shows CC101 results from row 1 in (A); second panel shows CC101 Δ*mutT* results from row 3 in (A); third panel shows CC101 Δ*mutT dnaE941* results from row 6 in (A); fourth panel shows CC101 Δ*mutT* Δ*mutY* results from row 5 in (A); last panel shows CC101 Δ*mutT* Δ*mutY dnaE941* from row 7 in (A).

To qualitatively monitor β-galactosidase enzyme activity in growing cultures, Xgal (0.5 mg/ml) was added to LB media (39), and a fluctuation test was performed. We seeded ∼200 cells to wells of a microtiter dish and monitored growth (OD_600_) and cleavage of Xgal (OD_615_) to observe the nature of the appearance of β-galactosidase activity during growth of the CC101 and Δ*mutT* compromised cultures (Figure 5A). As the burden of *mutT*-mediated mutagenesis is attenuated from Δ*mutT* to Δ*mutT* Δ*mutY* or Δ*mutT dnaE941* to Δ*mutT* Δ*mutY dnaE941*, the resulting cultures become less uniformly blue, and only some cultures turn blue whereas other cultures of the same strain remain clear. Indeed, in the CC101 Δ*mutT* Δ*mutY dnaE941* strain, the great majority of wells were clear and similar in appearance to the wild-type parent CC101 (as shown in the OD_615_ tracings; Figure 5A bottom panel). Therefore, although the amounts of 8-oxo-dGTP and 8-oxo-GTP remain the same in all these Δ*mutT* strains, the phenotypic consequences of these oxidized nucleotides are decreased; we emphasize that it is only the mutational consequences of 8-oxo-dGTP that are precluded, since DNA polymerase III and MutY do not incorporate 8-oxo-GTP nor act on 8-oxo-GTP incorporation, respectively.

When the time course of β-galactosidase enzyme activity in growing cultures is monitored (Figure 5B), the fluctuation distribution of β-galactosidase enzyme activity appearance becomes readily apparent. All Δ*mutT* wells exhibit an abrupt appearance of blue with the timing of color change varying from well to well. The timing of blue appearance becomes delayed and the numbers of wells that exhibit blue decreases as the mutational consequence of *mutT*-mediated mutagenesis is decreased. This is seen most clearly for the CC101 Δ*mutT* Δ*mutY dnaE941* strain when singular stochastic events in a few cultures produce markedly different end results from the majority of similar cultures (another independent experiment giving identical results is described in Supplementary Figure S3).

We observe a fluctuation type of distribution of β-galactosidase appearance in a growing culture, consistent with Lac^+^ revertant events, and not a gradual constant production of β-galactosidase that should arise from chronic incorporation of 8-oxo-GTP into the *lacZ* transcript that would transiently produce long-lived β-galactosidase molecules that would progressively accumulate (newly created revertant Lac^+^ mRNA being offset by dilution of β-galactosidase by cell division); moreover, this gradual accumulated appearance should be similar for all Δ*mutT* compromised cultures, since the amount of 8-oxo-GTP is the same for all such strains. However, we see no evidence for a steady increase in blue color. Therefore, these results are completely consistent with the quantitative assay of mutation and β-galactosidase enzyme activity (Figure 3 and Supplementary Table S2).

The CC101 *mutT* background has been used to great effect in the screening of mammalian cDNAs to identify genes that can prevent A:T → C:G mutations by cleaning up the nucleotide pool (44,45). For example, hMTH1 (44) and hNUDT5 (45), despite different substrate specificities, can replace MutT function: when the cDNA for each human protein was expressed in CC101 *mutT E. coli*, the mutator phenotype was completely suppressed (99.4 and 99.9% reduction in *lacZ* amber reversion rate, respectively). However, other studies have used the suppression of blue-ness in a CC101 Δ*mutT* background grown in LB plus Xgal, or in a more quantitative β-galactosidase enzyme activity assay, to screen for mammalian (39) or plant (46) cDNA functions that are considered to prevent transcriptional errors caused by oxidative damage. Although both DNA and RNA errors will contribute to the β-galactosidase enzyme activity in the *E. coli* cultures, it was assumed that the increased β-galactosidase enzyme activity in *mutT* cultures was due solely to RNA errors, an idea that we challenge based on our results. We suggest that the findings of Ishibashi *et al.* (39) and Yoshimura *et al.* (46) can be explained by DNA errors arising through incorporation of 8-oxo-dGTP during DNA replication since there is a perfect correlation between β-galactosidase enzyme activity and mutation rate that they observe in their cultures (suppression of mutation by the cDNA gives clear cultures indicative of low β-galactosidase enzyme activity; or incomplete suppression of mutation by the cDNA gives blue cultures indicating higher β-galactosidase enzyme activity). This eliminates any requirement for errors arising through incorporation of 8-oxo-GTP during transcription.

### MutT and transcription errors

The idea that persistent 8-oxo-GTP in the ribonucleotide pool, due to the absence of MutT function, can have phenotypic consequences for the cell is provocative (18,26). When we utilized an epigenetic switch system that can convert transient stochastic transcription error events into a heritable phenotype, via positive feed-back, we do not observe any increase in epigenetic switch frequency in a Δ*mutT* strain. When we decreased the mutational burden of Δ*mutT* in strain CC101 (although the levels of 8-oxo-dGTP and 8-oxo-GTP remain the same in all cells) we do not see any enhanced phenotypic effect attributable to RNA errors. Mutation fluctuation patterns become more pronounced as we decrease the mutational burden of *mutT*-mediated mutagenesis. Lac^+^ revertants can explain the increase in β-galactosidase enzyme activity in the Δ*mutT* cultures. Therefore, our results suggest that 8-oxo-dGTP can have ∼500× more relative DNA mutational impact during DNA replication than the RNA mutational impact of 8-oxo-GTP during RNA transcription.

It has been estimated that the content of 8-oxo-G in the DNA of *mutT* cells due to the incorporation of 8-oxo-dGTP is about four per 10^6^ guanine residues (47). If the relative amounts of 8-oxo-dGTP to dGTP and 8-oxo-GTP to GTP in the pools are the same, and the propensity of DNA polymerase to incorporate 8-oxo-dGTP is the same as the propensity of RNA polymerase to incorporate 8-oxo-GTP relative to the non-oxidized nucleotide, then there should also be about four 8-oxo-G per 10^6^ guanine residues in total RNA (26). Concerning the mutational consequences of such oxidized nucleotide incorporation, one also needs to consider the specificity of incorporation by the polymerase opposite A (mutagenic outcome) or C (non-mutagenic outcome) bases residing in the template. While it has been shown that *E. coli* DNA polymerase III can use 8-oxo-dGTP as efficiently as dGTP (14), 8-oxo-GTP is incorporated into RNA by *E. coli* RNA polymerase at a rate of only 4% of that for GTP (48). Moreover, while the incorporation of 8-oxo-GTP opposite template A and C occurs with similar efficiencies with RNA polymerase (49), DNA pol III incorporates 8-oxo-dGTP 20× more efficiently opposite template A compared with template C (15). Therefore, 8-oxo-dGTP should have ∼500× more relative DNA mutational impact during DNA replication than the RNA mutational impact of 8-oxo-GTP during RNA transcription. Due to the lower efficiency of RNA polymerase 8-oxo-GTP incorporation, and the lower mutagenic potential of such incorporation, we suggest that *mutT*-mediated RNA errors during transcription do not significantly increase the rate of ∼10^−5^ transcription errors per residue observed in wild-type *E. coli* (1,2). In fact, the level of 8-oxo-G in cellular RNA increases from one 8-oxo-G per 10^5^ guanine residues to ten 8-oxo-G per 10^5^ guanine residues during exposure to 5-mM H_2_O_2_, a treatment that kills 50% of the cells (50). This measurement takes into account both 8-oxo-G incorporation into RNA and also oxidation of G after incorporation into RNA. Thus, the difference in the relative mutational impact of 8-oxo-dGTP and 8-oxo-GTP in the absence of MutT function would explain the results we observe in this study, namely, Δ*mutT* creates a strong DNA mutator but does not equally create a strong RNA mutator.

Therefore, we find little evidence to support the idea that the absence of MutT function at the level of transcription produces any significant transient (phenotypic suppression) or heritable (epigenetic switching) consequences for the phenotype of the cell, and instead suggest that the observed increase in β-galactosidase levels in *mutT* strains is due to *mutT*-mediated mutagenesis. Although we have shown that transient transcription errors can have phenotypic consequences for the cell (5,6), and we continue to assess RNA mutator candidates, we do not find the Δ*mutT* situation to act as an RNA mutator.

A high level of reactive oxygen species can be detrimental for cell survival because of damage to DNA, RNA, protein and lipids. Whereas the removal of oxidized dGTP by MutT is critical to reduce DNA replication errors, it remains unclear how the persistence of oxidized GTP in the nucleotide pool can trigger aminoglycoside antibiotic cytotoxicity (20) or increase protein mistranslation in bacteria (19).

## SUPPLEMENTARY DATA

Supplementary Data are available at NAR Online.

SUPPLEMENTARY DATA
