# Supplementary material for: Removal of 8-oxo-GTP by MutT hydrolase is not a major contributor to transcriptional fidelity
Source: Nucleic Acids Res. 2014 Oct 7;42(19):12015–26. doi: 10.1093/nar/gku912 (PMC4231768; doi:10.1093/nar/gku912)
Supplement: SUPPLEMENTARY DATA [file supp_42_19_12015__index.html]

Removal of 8-oxo-GTP by MutT hydrolase is not a major contributor to transcriptional fidelity — SUPPLEMENTARY DATA 

# Removal of 8-oxo-GTP by MutT hydrolase is not a major contributor to transcriptional fidelity

## SUPPLEMENTARY DATA

**Files in this Data Supplement:**

- SUPPLEMENTARY DATA
