# Supplementary material for: Removal of 8-oxo-GTP by MutT hydrolase is not a major contributor to transcriptional fidelity
Source: Nucleic Acids Res. 2014 Oct 7;42(19):12015–26. doi: 10.1093/nar/gku912 (PMC4231768; doi:10.1093/nar/gku912)
Supplement: SUPPLEMENTARY DATA [file supp_gku912_NAR-02350-D-2014_revised.pdf]

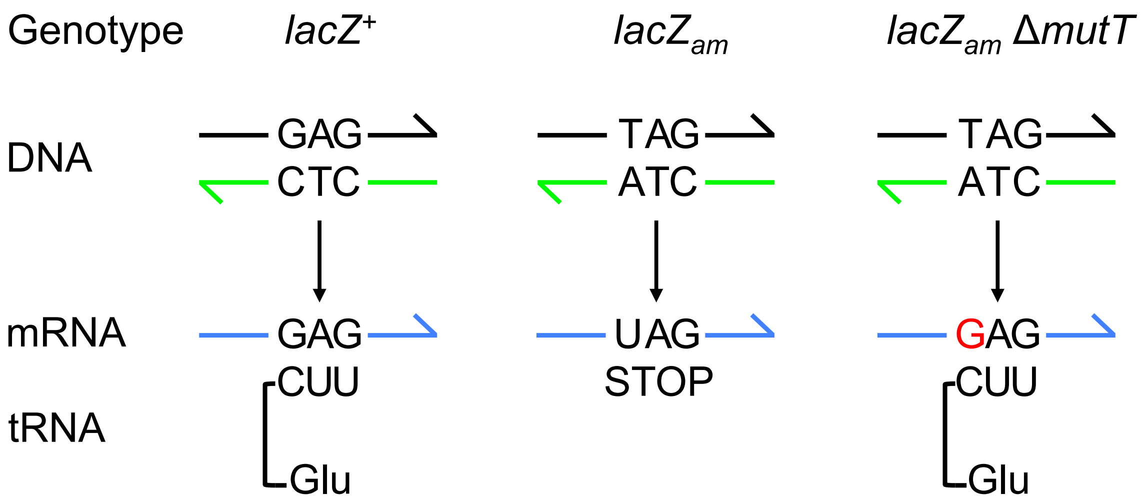

Figure S1. *lacZ*$_{am}$ leakiness and 8-oxo-GTP. 8-oxo-GTP can be incorporated by RNA polymerase opposite template A during transcription. The CTA in the transcribed strand of the *lacZ* amber mutant would then be copied to 8-oxo-GAG (which would pair with the UUC glutamic acid anticodon) rather than to a UAG stop codon in the nascent transcript. Therefore, such a rare transcript would code for wild-type β-galactosidase monomers and generate a transient burst of β-galactosidase activity in the cell that would subside when the transcript is degraded and the β-galactosidase activity would eventually dissipate through dilution when cell division occurs. Arrows indicate 5` ➔ 3` polarity of the DNA/RNA chain. Black arrows, non-transcribed DNA strand; green arrows, transcribed DNA strand; blue arrows, nascent mRNA strand; red G represents 8-oxo-GTP. It has been noted that *E. coli* does not possess a tRNA with the CUC glutamic acid anticodon (35).

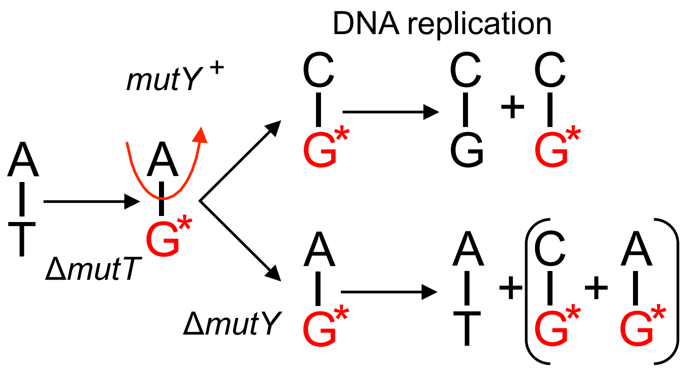

Figure S2. MutY repair is mutagenic in Δ *mutT* strains of *E. coli*. During DNA synthesis, in the absence of MutT function, 8-oxo-dGTP (G*) can be incorporated opposite the template adenine residue. In the presence of MutY function, the adenine residue will be removed from A:G* mispairs (red arrow) ultimately resulting in A:T ➔ C:G transversions. In the absence of MutY function, subsequent DNA replication at the A:G* mispair will restore an A:T pair in one chain, and C or A residues will be incorporated opposite the G* site leading to A:T ➔ C:G transversions or restoration of the A:T pair, respectively, in the other chain upon subsequent replication. Therefore, the absence of MutY function will reduce the number of A:T ➔ C:G transversions in a Δ*mutT* strain by ~75% compared to a *mutY*$^{+}$ Δ*mutT* strain, if C and A are incorporated with equal efficiency across from template G*. Indeed, we observe a 70% reduction in such events, similar to the 71% reduction observed by Vidmar & Cupples (36). Figure modified from Vidmar & Cupples (36).

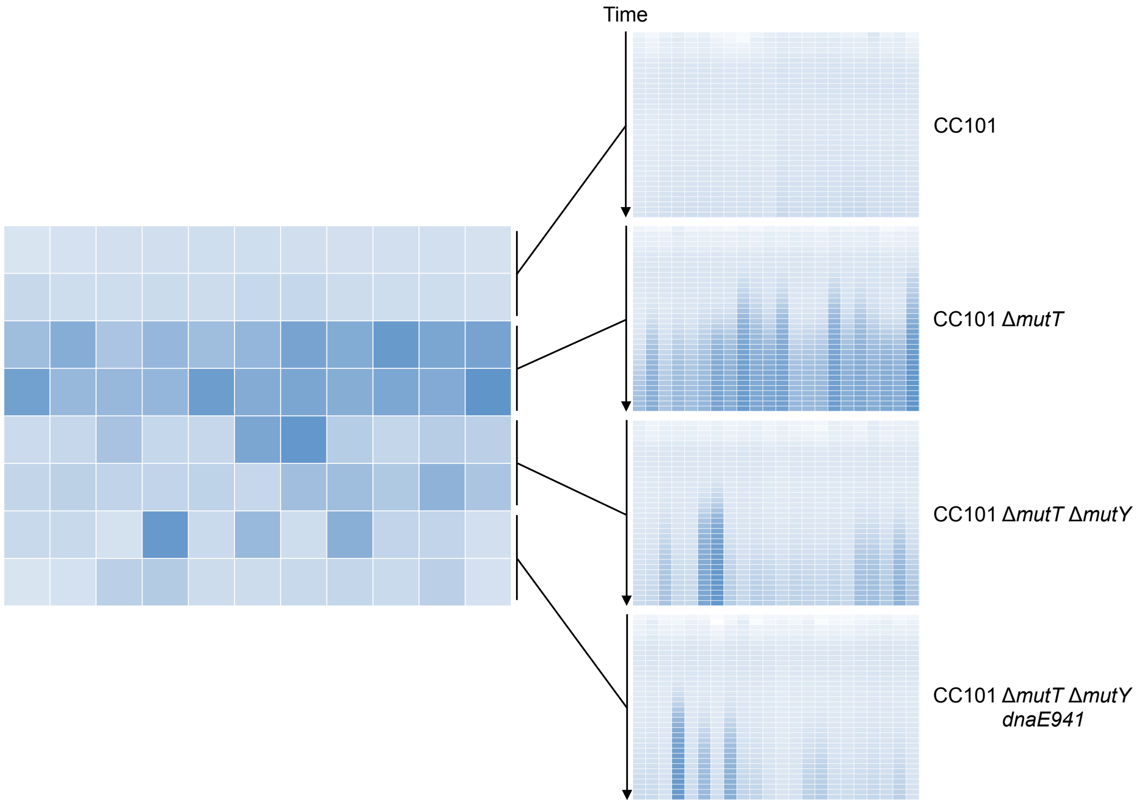

Figure S3. A fluctuation test analysis of Lac^+ revertants in CC101 and Δ *mutT* compromised strains. This is an independent repeat of a similar experiment described in Figure 5. The panel on the left is a heat map representation of the OD_{615} scans showing the fluctuation nature of the observance of Lac^+ mutation after 45 hours growth and incubation at 37° (see Figure 5). The panels on the right are heat map representations of the OD_{615} scans over time showing the fluctuation nature of the observance of Lac^+ mutation (see Figure 5). Each square of the heat map on the left represents an independent culture growing in a single well of a microtiter dish. Each column of the heat maps on the right represents the corresponding culture over time (indicated by the black arrows); each row represents normalized OD_{615} readings, at hourly intervals, starting at hour 10 and ending at hour 45.

Table S1. Sequences of smFISH probes.

Probe sequences (5' → 3')	Probe name
ctgaattgactctcttccgg	MW_lacI_1
ttactggtttcacattcacc	MW_lacI_2
catactctgcgacatcgtat	MW_lacI_3
cgcgggaaacggtctgataa	MW_lacI_4
ttttcgcagaaacgtggctg	MW_lacI_5
cgcggttgggaatgtaattc	MW_lacI_6
ccaatcagcaacgactgttt	MW_lacI_7
agatttaatcgccgcgacaa	MW_lacI_8
cttcgttctaccatcgacac	MW_lacI_9
cgttgcgcgagaagattgtg	MW_lacI_10
gatagttaatgatcagccca	MW_lacI_11
taacgccggaacattagtgc	MW_lacI_12
atactgttgatgggtgtctg	MW_lacI_13
taccgtcttcatgggagaaa	MW_lacI_14
aatgcgaccagatgctccac	MW_lacI_15
ctaacagcgcgatttgctgg	MW_lacI_16
gcgccgagacagaacttaat	MW_lacI_17
gagatatttatgccagccag	MW_lacI_18
cgctatcggctgaatttgat	MW_lacI_19
tgaaaaccggacatggcact	MW_lacI_20
attcagcatttgcatggttt	MW_lacI_21
atctgatcgttggcaaccag	MW_lacI_22
cactaccgagatatccgcac	MW_lacI_23
tgtcttcggtatcgtcgtat	MW_lacI_24
gttgacggcgggatataaca	MW_lacI_25
agcaggcgaaaatcctgttt	MW_lacI_26
tgagagagttgcagcaagcg	MW_lacI_27
agacgggcaacagctgattg	MW_lacI_28
agggtggtttttcttttcac	MW_lacI_29
gagaggcggtttgcgtattg	MW_lacI_30
gccagctgcattaatgaatc	MW_lacI_31
ctttccagtcgggaaacctg	MW_lacI_32

Table S2. Lac$^+$ mutation frequencies and β-galactosidase enzyme activities

Strain	Lac$^+$ revertants per 10^8 cells	β-galactosidase activity (Miller units)
CC101 *mutT*$^+$	0.55 ± 0.24	0.00315 ± 0.00025
CC101 Δ*mutT*	2060 ± 324	0.01687 ± 0.00888
CC101 Δ*mutT* Δ*mutY*	801 ± 101	0.01211 ± 0.00194
CC101 Δ*mutT* Δ*mutY* *dnaE941*	284 ± 61	0.00862 ± 0.00168

Frequencies are means (± SD) for nine or ten independent cultures per strain (see Figure 3); the mutation jackpot observed in the Δ*mutT* background is omitted from this analysis.
